# Pricing Resources in LTE Networks through Multiobjective Optimization

**DOI:** 10.1155/2014/394082

**Published:** 2014-01-02

**Authors:** Yung-Liang Lai, Jehn-Ruey Jiang

**Affiliations:** ^1^Department of Computer Science and Information Engineering, Taoyuan Innovation Institute of Technology, Taoyuan, Jhongli 32001, Taiwan; ^2^Department of Computer Science and Information Engineering, National Central University, Taoyuan, Jhongli 32001, Taiwan

## Abstract

The LTE technology offers versatile mobile services that use different numbers of resources. This enables operators to provide subscribers or users with differential quality of service (QoS) to boost their satisfaction. On one hand, LTE operators need to price the resources high for maximizing their profits. On the other hand, pricing also needs to consider user satisfaction with allocated resources and prices to avoid “user churn,” which means subscribers will unsubscribe services due to dissatisfaction with allocated resources or prices. In this paper, we study the pricing resources with profits and satisfaction optimization (PRPSO) problem in the LTE networks, considering the operator profit and subscribers' satisfaction at the same time. The problem is modelled as nonlinear multiobjective optimization with two optimal objectives: (1) maximizing operator profit and (2) maximizing user satisfaction. We propose to solve the problem based on the framework of the NSGA-II. Simulations are conducted for evaluating the proposed solution.

## 1. Introduction

The 3rd generation partnership project (3GPP) long-term evolution (LTE) technology is one of the major candidates of the fourth generation (4G) wireless communication systems [1]. It offers versatile mobile services using different numbers of resources and enables operators to provide subscribers or users with differential quality of service (QoS) for maximizing subscriber satisfaction. The LTE operators seek the deployment of spectrum-efficient, ubiquitous, always-on, and interoperable mobile broadband wireless access, whose goal is to provide peak data rates of 100 Mbps for high-mobility subscribers and 1 Gbps for low-mobility subscribers [2].

Due to scarcity of resources (e.g., spectrum) in the LTE network, the resources are usually costly. The operators invest huge funds in capital expenditure (CAPEX) and operational expenditure (OPEX) for spectrum licensing and infrastructure construction and management [3], in order to reserve enough resources for subscribers to boost their satisfaction. On one hand, LTE operators need to price the resources high for maximizing their profits. On the other hand, pricing also needs to consider user satisfaction with allocated resources and prices to avoid the “user churn” [4], which means subscribers will unsubscribe services due to dissatisfaction with allocated resources or prices. It is important to study how to price the resources for maximizing the operator profit and maximizing the subscriber satisfaction at the same time in LTE networks.

This paper investigates the pricing resources with profit and satisfaction optimization (PRPSO) problem in the LTE networks to simultaneously maximize the operator profit and subscriber satisfaction. The problem is modelled as a multiobjective problem with two conflicting objectives: (1) maximizing operator profit and (2) maximizing user satisfaction. The PRPSO problem is modeled on the resource block allocation model defined in the 3GPP LTE standard [2]. For an LTE operator, the solutions of the PRPSO problem are helpful for analyzing realistic impacts of investment in spectrum, since the PRPSO problem is formulated on the *resource blocks*, which are the units of allocation mechanism for allocating spectrum resources for subscribers and are the units for providing enough quality of services in the LTE network.

Due to the hardness of solving this problem, we develop a heuristic genetic optimization algorithm, called the PRPSO algorithm, to find the solution based on the nondominated sorting genetic algorithm (NSGA-II) in [5]. For an analyst of pricing resources in LTE networks, the solutions found by the PRPSO algorithm are useful for analyzing the subscribers' satisfaction and the operator's profit, based on subscribers' satisfaction models and costs for acquiring spectrum. The optimal solutions found by the PRPSO algorithm are the Pareto fronts in the multiobjective decision theory. The Pareto fronts are a set of choices that are nondominated by other choices, which are helpful for making tradeoff decisions to achieve two conflicting objectives, subscribers' satisfaction and operator's profits, in LTE networks.

Some optimization studies are proposed for resource pricing, resource reservation, and load balancing in LTE networks. Huang et al. in [6] proposed an adaptive call admission control and resource (or bandwidth) reservation scheme using fuzzy logic control and particle swarm optimization (PSO) for 4G networks. Huang et al. in [7] proposed a resource (or bandwidth) reservation mechanism for neighboring 4G cells based on grey prediction theory and swarm intelligence. Dixit et al. in [8] studied the dynamic pricing problem on maximizing operator revenue in LTE networks. However, the problem of reserving and pricing LTE wireless resources to maximize both the operator profit and subscriber satisfaction is not fully studied.

The rest of this paper is organized as follows. In Section 2, the PRPSO problem is formulated. The proposed heuristic genetic PRPSO algorithm to solve the problem is introduced and evaluated in Sections 3 and 4, respectively. And finally, some concluding remarks are drawn in Section 5.

## 2. Modeling and Problem Formulation 

In this section, we introduce the resource allocation model, the user satisfaction model, and the PRPSO problem in LTE networks.

### 2.1. Resource Allocation Model in LTE Networks

**Figure 1:**
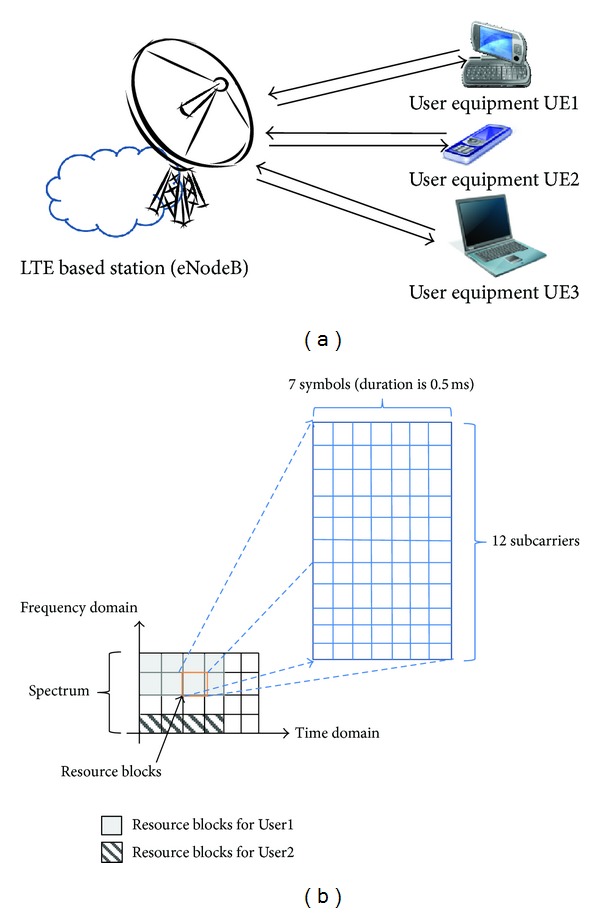
Illustration of services controlled and resources allocated by an LTE based station (eNodeB) in the LTE network. (a) UE with differential QoS requirements of services and (b) resource blocks allocated for a UE by a eNodeB in the frequency domain and time domain.

The LTE network uses an IP-based network architecture to provide voice and data services. Based on the architecture, the operator can design and sell the products by combining different QoS services. The LTE air interface uses orthogonal frequency division multiplexing (OFDM) with advanced antenna techniques to transmit voice and data simultaneously [2]. The scheduler in the base station (called eNodeB or eNB) is responsible for dynamically scheduling the LTE air interface in both the downlink and uplink directions for subscribers' user equipment (UE). As shown in Figure 1(a), much UE accesses an eNB at the same time, and the eNB needs to provide differential services for the UEs.

The LTE standard provides a diversity of classes of QoS services [9]. A traffic class or a QoS class is defined according to the restrictions and the limitations of the radio interface. Based on the traffic sensitivity to the packet delay, four classes are defined: the *conversational*, *streaming*, *interactive*, and *background* classes. The conversational class is meant for the traffic that has a high sensitivity to delay (e.g., VoIP), while the background class deals with the traffic that has a low sensitivity to delay (e.g., background downloading files or sending emails). The streaming class is real time based and can preserve time relation (variation) between information entities of streams (say video streams). The interactive class is best effort based and follows a request-response pattern in applications, such as web browsing.

In LTE networks, a set of resource blocks is allocated to a subscriber [10] to provide the services. The more resource blocks are allocated to the subscriber, the better experience of service is, and thus the satisfaction is increased. As shown in Figure 1(b), the downlink (eNB to UE) and uplink (UE to eNB) in the air interface are divided into a number of 15 kHz subchannels in the frequency domain and a number of 0.5 ms time slots in the time domain. The resource block (RB) is the main unit used to schedule transmissions over the air interface (refer to Figure 1(b)). An RB contains 12 contiguous subchannels and 7 symbols (duration is 0.5 ms). In general, a number of RBs are allocated to an UE according to its quality of service (QoS) [11]. Statistically, the more the resource blocks are allocated to the UE of a subscriber, the more the subscriber is satisfied with the service. In Figure 1(b), the number of allocated resource blocks of User 1 is higher than that of User 2, which implies that the satisfaction of the User 2 is higher than User 1.

The resource block based model is more accurate in analysing the channel resources used in the LTE network, since it considers not only the cost of transmitting data but also the overhead cost (such as retransmissions when packets are corrupted). It is notable that most of studies on pricing are based on the received usage based model to charge subscribers based on the amount of received packets, which do not include the overhead cost.

### 2.2. User Satisfaction Model

An operator allocates or reserves resources to subscribers to provide them with differential QoS levels. The satisfaction of subscribers is important to the operator. Without sufficient resources allocated to the services, subscribers will feel dissatisfied. For example, the acceptable one-way (speaker's mouth to listener's ear) delay of voice communication for VoIP applications recommended by ITU [12] is at most 150 ms. The subscriber of VoIP service will feel dissatisfied if transmission delay is more than 150 ms due to the insufficiency of allocated resources. Dissatisfied subscribers may unsubscribe some services, which will damage the operator's profits. They may unsubscribe all services and migrate to another operator, causing the “user churn” problem reported in [4].

Lin et al. in [13] proposed a method to approximate the subscriber's satisfaction with the allocated resources by a sigmoid function. In this paper, we also adopt the sigmoid function to model the user satisfaction. The sigmoid function is useful for modeling natural processes or system learning curves, since it can represent a history dependent progression approaching a limit. It depends on a random variable *x* to represent the occupation of resources for the subscriber. The sigmoid function is formulated as follows:

(1) $$\begin{array}{c} \Psi \left(x\right)=\frac{1}{\left(1+e^{-\alpha (x-\beta )}\right)}, \end{array}$$

where *α* and *β* are the steepness and the middle of the curve, respectively.

**Figure 2:**
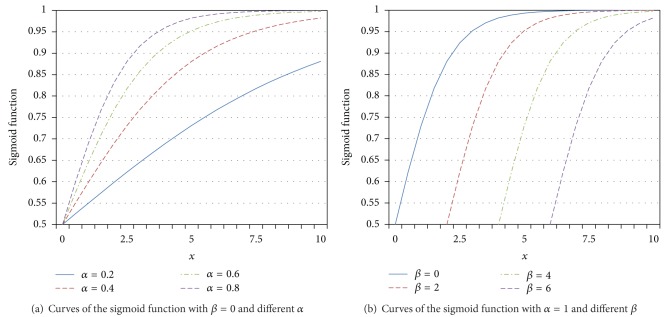
Curves of the sigmoid functions.

Figure 2 plots curves of the sigmoid functions, where *α* stands for the sensitivity and *β* is the median value of the satisfaction curve. As shown in Figure 2(a), the curve is with higher steepness as *α* is higher. As shown in Figure 2(b), the starting point of the curve is farther away from zero as *β* is higher.

In subscriber's point of view, the value of *α* indicates the subscriber's sensitivity to the degradation of service, while *β* indicates the acceptable level for the service. It is remarkable that *β* decides when the satisfaction starts to increase and *α* decides how fast the satisfaction increases.

### 2.3. The Pricing Resources with Profit and Satisfaction Optimization (PRPSO) Problem

We formulate the pricing resources with profit and satisfaction optimization (PRPSO) problem in this subsection. The main goals of the problem are (1) to maximize the operator's profit and (2) to maximize the users' satisfaction. The formulation is based on a fixed period of time *T*, say 1 day, 1 month, 2 months, 1 year, and so on.

The first goal is to maximize the operator's profit. Equation (2) is used to formulate the operator's profit, *P*, which consists of two factors, revenue from subscribers (RS) and cost of spectrum (CS). Below we explain the meaning of (2) and describe some assumptions and notations used in it.

We assume an operator has a set of spectrum segments (notated by Φ). For segment *i* ∈ Φ, *B*_*i*_ represents the quantity of units occupied in segment *i*, and *C*_*i*_ represents the cost per unit of segment *i*. Thus, the operator pays CS = ∑_*i*∈Φ_ *B*_*i*_ *C*_*i*_ for acquiring the spectrums for period *T*. We also assume the operator sells a set of services (notated by *Ω*) to subscribers. By statistics or by predictions, the operator totally allocates *Q*_*s*_ resource blocks for each service *s* ∈ *Ω* over period *T*. Therefore, if the subscribers pay the price *P*_*s*_ for each resource block allocated to service *s* for period *T*, the operator has revenue RS = ∑_*s*∈*Ω*_ *Q*_*s*_ *P*_*s*_ from the subscribers. For example, assume a service *Sa* is averagely allocated 100 resource blocks per day and the price of each resource block allocated to service *Sa* is 1.2 dollar. It means *Q*_*sa*_ is 100 resource blocks and *P*_*sa*_ is 1.2 dollar per day; thus the revenue is 120 dollar per day for service *Sa*. Consider

(2) $$\begin{array}{c} P=\text{RS}-\text{CS}=\sum_{s\in \Omega }^{}Q_{s}P_{s}-\sum_{i\in \Phi }^{}B_{i}C_{i}. \end{array}$$

*P*_*s*_ denotes the price per resource block allocated to service *s*. In reality, *P*_*s*_ is a bounded price variable and *P*_*s*_ ∈ [*P*_min_, *P*_max_] as shown in

(3) $$\begin{array}{c} P_{\min}\leq P_{s}\leq P_{\max}. \end{array}$$

The second goal is to maximize the subscribers' satisfaction. Equation (4) is used to formulate the satisfaction per paid price *U*. In (4), *ψ*_*s*_(*Q*_*s*_) is used to formulate the satisfaction for service *s*; it is a sigmoid function of *Q*_*s*_, the number of resource blocks allocated to service *s*. Note that *Q*_*s*_ *P*_*s*_ is the price a subscriber pays for using service *s*. Therefore, *ψ*_*s*_  (*Q*_*s*_)/*Q*_*s*_ *P*_*s*_ is the satisfaction per unit of paid price for service *s*. *U* is hence the overall subscribers' satisfaction per unit of paid price

(4) $$\begin{array}{c} U=\sum_{s\in \Omega }^{}\psi_{s}\frac{\left(Q_{s}\right)}{Q_{s}P_{s}}. \end{array}$$

Given *Q*_*s*_, *B*_*i*_, *C*_*i*_, and *ψ*_*s*_(*Q*_*s*_), ∀  *i* ∈ Φ, *s* ∈ *Ω*, the PRPSO problem is to find a price set PS for maximizing both the operator's profit and subscribers' satisfaction, defined as

(5) $$\begin{array}{c} \text{Maximize}\;P,\\ \text{Maximize}\;U. \end{array}$$

Now, we discuss some issues of estimating parameters in the PRPSO problem. The PRPSO problem is an optimization problem to decide prices, based on given information. The quantity *Q*_*s*_ of resource blocks allocated to service *s* is possibly estimated from historic usage of resource blocks allocated to service *s* over the fixed period of time *T*. The per-unit cost *C*_*i*_ of spectrum segment *i* is also possibly estimated as the average cost of acquiring and managing spectrum over the time period *T*. When the solutions of PRSP problem are found, the output prices are also on the basis of time period *T*. For example, if *Q*_*s*_ and *C*_*i*_ are estimated over the period of one month, the prices are on the basis of one month. It is also notable that the parameters *α* and *β* of *ψ*_*s*_ can be adjusted according to subscribers' experiences to shape the sigmoid function properly.

## 3. The Pricing Resources with Profit and Satisfaction Optimization (PRPSO) Algorithm

In this section, we present our multiobjective pricing algorithm, called the PRPSO algorithm, to solve the pricing resources with profit and satisfaction optimization (PRPSO) problem. The proposed PRPSO algorithm is based on an evolutionary genetic algorithm (GA) approach, which is used to heuristically find the solutions of optimization problems. The GA approach is to mimic natural selection in the biology, where individuals with higher fitness can survive to next generation [14].

In the GA approach, the population (a set of individuals or solutions) is randomly generated in the initial step. Then, the population evolves in the generation loop for MAX_GEN times. In each generation, fundamental operations, such as selection, crossover, and mutation are used to generate individuals into the next generation. When the generation loop is terminated, the solution is made by decoding the best individuals in the decode step.

Based on the above steps and based on the nondominated sorting genetic algorithm II (NSGA-II) in [5], we design the PRPSO algorithm for finding good solutions to the PRPSO problem. The basic idea of the NSGA-II algorithm is to find, from the solutions of the current and the next generations, the optimal front (called Pareto front), which is the set of nondominated feasible solutions (or front points) that are not dominated by any others. It is noted that a solution *x* is said to dominate another solution *y*, if and only if *x* is better than *y* in at least one evaluation of objectives and *x* is not worse than *y* in all evaluations of objectives.

*N* solutions in the Pareto front are selected to evolve, as the population is assumed to be of size *N* for each generation. If the first-found optimal front (or call the first optimal front) has less than *N* front points, then the second optimal front should also be found. The second optimal front is the set of nondominated feasible solutions over all population members except for those in the first optimal front. If the first and the second optimal fronts totally have less than *N* members, then the third optimal front should be found further and so on. Not all the front points in the last-found optimal front are selected. They are in practice selected according to the fitness (i.e., the nondomination) and the spread of solutions so that the optimal front found in the final generation will have better convergence near the true Pareto front. It is noted that the notion of crowding distance is used for evaluating the degree of spread of solutions.

Now, we introduce how to evaluate an individual of a population in the proposed algorithm. Each individual (say *x*) in the population has two attributes: (1) nondomination *x*_rank_ and (2) crowding distance (*x*_*c*_dist_), where *x* has rank 1 (or 2, 3,…) if it belongs to the 1st (2nd, 3rd,…) optimal front, and the crowding distance is the summation of distances between *x* and two adjacent individuals in every evaluation of objectives (please refer to [5] for the details of crowding distance calculation). A partial order ≺_*n*_ is defined between two individuals *x* and *y* in

(6) $$\begin{array}{c} x{≺}_{n}y,\;\text{if}\{\begin{array}{cc} x_{\mathrm{rank}}<y_{\mathrm{rank}} & \\ x_{\mathrm{rank}}=y_{\mathrm{rank}},\;\;x_{c\_\mathrm{dist}}>y_{c\_\mathrm{dist}}. & \end{array} \end{array}$$

In (6), between two individuals or solutions with differing nondomination ranks, we prefer the solution with the lower (better) rank. Otherwise, if both solutions belong to the same front, then we prefer the solution that is located in a less crowded region.

**Figure 3:**
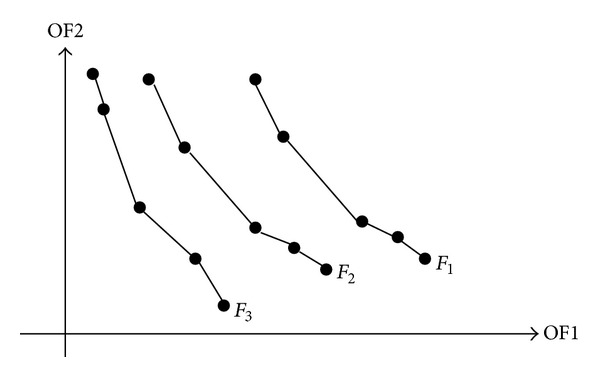
Illustration of a front set *F*, where *F* = {*F*_1_, *F*_2_, *F*_3_}. Each point represents one feasible solution in one front in the 2-dimensional space. *F*_1_ (resp., *F*_3_) is best (resp., worst) front, and the solutions in *F*_1_, *F*_2_, and *F*_3_ have the nondomination rank of 1, 2, and 3, respectively.

The PRPSO algorithm runs generation by generation. In each generation, a front set *F* = {*F*_1_, *F*_2_, …, *F*_*r*_} is produced from both populations of the current and the previous generations, where *F*_1_, *F*_2_, …, *F*_*r*_ are the 1st, 2nd,…, *r*th optimal fronts and *r* is the maximum number of fronts to be accommodate in a population of size *N* (i.e., |*F*_1_| + |*F*_2_| + ⋯|*F*_*r*−1_| < *N* and |*F*_1_| + |*F*_2_| + ⋯|*F*_*r*−1_| ≥ *N*). As shown in the example in Figure 3, there are three fronts (*F*_1_, *F*_2_, and *F*_3_) produced on the two dimensional space, where the two dimensions correspond to the two objective functions OF1 and OF2. Front *F*_1_ is the set of solutions that are not dominated by any others. Each solution in front *F*_*i*_ is not dominated by any solution in front *F*_*j*_, for all *j* > *i* ≥ 1. The optimization goals in the PRPSO problem are to maximize the OF1 (i.e., profit: **P**, defined in (3)) and OF2 (i.e., satisfaction: **U**, defined in (4)), so an optimal front is the farthest from the origin point.

Since the populations are generated from the parents with the best finesses of the previous generation, the goodness of populations will be improved after some generations. In addition, the diversity of solutions is kept by the crowding distance so that the solutions widely spread. In this way, when the algorithm terminates, the returned optimal front *F*_1_ will be very close to the real Pareto front.

The pseudocode of the PRPSO algorithm is shown in Algorithm 1. Initially, the generation counter *t* is 0 and the population *P*_*t*_ is randomly generated, where a member in *P*_*t*_ is an individual (or a solution) consisting of the price variable, which is a vector for multiple service cases. An offspring population *Q*_*t*_ is set as empty initially.

**Figure 4:**
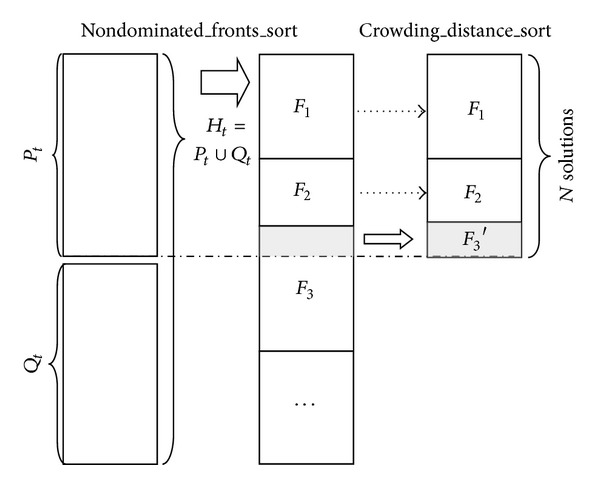
Procedures of generating new population *P*_*t*+1_ from *P*_*t*_ and *Q*_*t*_, where *P*_*t*_ is the parent population and *Q*_*t*_ is the child population.

As illustrated in Algorithm 1, in step S1, we set *H*_*t*_ to be the union of *P*_*t*_ and *Q*_*t*_. The step S1 is also illustrated in Figure 4. In step S2, the algorithm evokes the Nondominated_Fronts_Sort (*H*_*t*_, **P**, **U**) subroutine to sort solutions according to their nondomination ranks to have a front set *F* = {*F*_1_, *F*_2_, …, *F*_*r*_}.

The step S3 is to set the population *P*_*t*+1_ to be empty and set the counter *i* to be 1 before the algorithm enters the loop in step S4. The step S4 is to insert the nondominated solutions into *P*_*t*+1_. The step S5 is to generate a sorted *F*_*i*_ by the crowding distance in the descending order. The step S6 is to insert the most widely spread (*N* − |*P*_*t*+1_|) solutions using the crowding distance values in the sorted front *F*_*i*_ into the *P*_*t*+1_.

The step S7 is to create new offspring population *Q*_*t*+1_ from *P*_*t*+1_ by mutation and crossover operations, where the size of *Q*_*t*+1_ is *N*. In step S8, the algorithm checks whether the maximum generation is reached. If the generation counter *t* is less than the maximum value (MAX_GEN), then *t* is increased by 1 and then the algorithm goes to step S1; otherwise, the algorithm terminates.

## 4. Evaluation

In this section, we evaluate the effectiveness of proposed algorithm. The simulation is conducted by the simulator developed on Matlab [15]. The simulation of the proposed algorithm is conducted with following setting in Table 1.

The parameters used in the simulation are listed as follows. The initial population is created using a uniform random distribution. The population size is 15 · |*X*|, where |*X*| is the number of prices, each of which corresponds to a service. The price is a real number, whose range is from 1 to 2. We set the Pareto fraction as 0.35, which means the algorithm will try to limit the number of individuals in the current population that are on the Pareto front to 35 percent of the population size.

In the simulation, two conditions are used to determine whether to stop the algorithm execution. In Condition-1, the algorithm stops when the maximum number of generations (MAX_GEN) is reached, where the MAX_GEN is 200 · |*X*|. In Condition-2, the algorithm stops if the average change in the spread of the Pareto front over the “StallGenLimit” generations is less than the tolerable threshold (TolerateThresold). The algorithm stops when either of the conditions is satisfied.

### 4.1. Evaluation of Tradeoff Relationship of Two Conflicting Objectives

**Figure 5:**
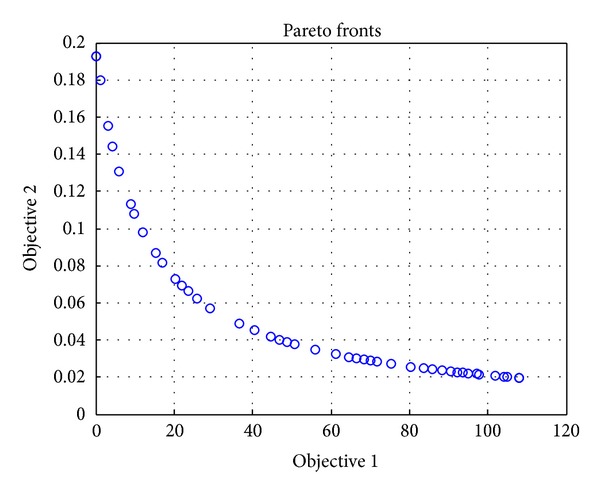
Results of pricing under two conflicting objectives, where Objective 1 is the operator's profit and Objective 2 is subscribers' satisfaction.

We first simulate the proposed algorithm in the basic setting, which is to decide price variables for three services, for the evaluation of tradeoff relationship of two conflicting objectives. The simulation results are shown in Figure 5, where Objective 1 is operator's profit and Objective 2 is subscribers' satisfaction per unit of paid price. Several front points are plotted in Figure 5, which form the Pareto front of the multiobjective optimization theory. Each point has two values, which are the operator's profit and the subscriber's satisfaction. As shown in Figure 5, the lower profit implies the higher satisfaction, while the higher profit implies the lower satisfaction. In summary, it is impossible to increase the profit and satisfaction at the same time, and thus there is tradeoff between the two objectives: the operator's profits and subscribers' satisfaction.

### 4.2. Evaluation of Impacts of Raising Prices of Two Conflicting Objectives

In this section, we study the effectiveness of raising prices of the services. We add into (2) an additional variable for controlling price raising factor *δ* to have (7), where the price raising factor *δ* is 1,1.2,…, 2,

(7) $$\begin{array}{c} P=\sum_{s\in \Omega }^{}Q_{s}\left(\delta \cdot P_{s}\right)-\sum_{i\in B}^{}B_{i}C_{i}. \end{array}$$

**Figure 6:**
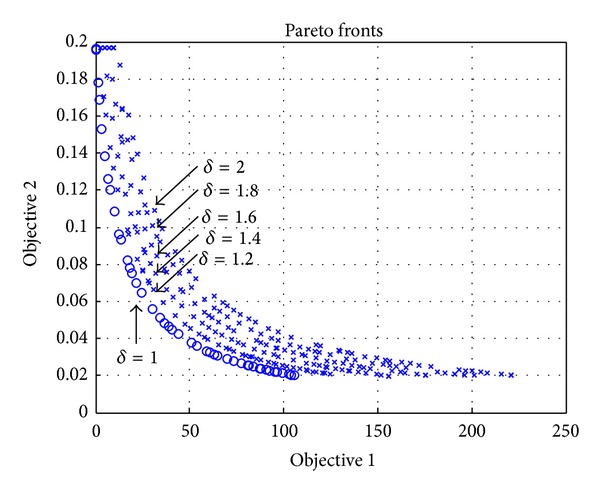
Evaluation of the effect of raising prices by adjusting the price raising factor *δ*.

As shown in Figure 6, the maximum values of profit (Objective 1) of Pareto fronts move to the right, if the price raising factor *δ* is increased. It reflects the effectiveness of raising prices to increase the profit.

The solutions found by the proposed algorithm are stable, since the results do not fluctuate along the curves, as shown in Figure 6. Moreover, the effect of raising prices can be easily observed in the results. For example, the maximal profit of original curve (*δ* = 1) is 108, and the maximal profit of adjusted curve (*δ* = 2) is 214, as shown in the Figure 6. Therefore, the maximum profit is almost doubled, meaning the effect of raising price is obvious.

### 4.3. Evaluation of Impacts of Raising Prices of Two Conflicting Objectives

**Figure 7:**
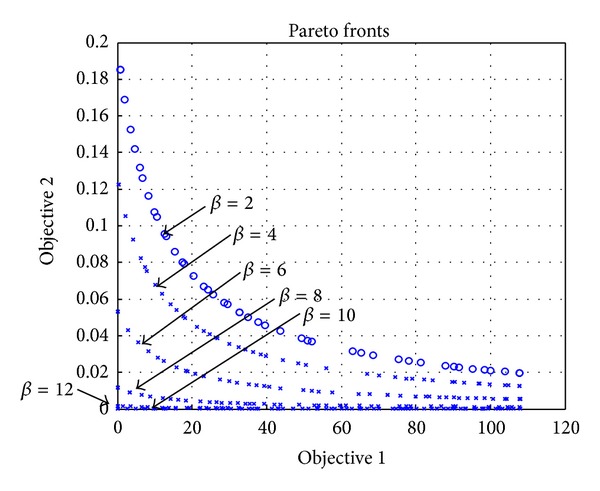
Evaluation of the effect of changing sensitivity of satisfaction on services.

We study in this section the effect of changing of the median value (*β*) of the satisfaction of services. We set the median value (*β*) as 2, 4,…, 12 in order to analyze the corresponding results. As shown in Figure 7, if *β* is increased, satisfaction is decreased. This is because a subscriber starts to feel satisfied only after a lot of resources are allocated to him/her for the cases of higher *β* values.

The results show that the difference of satisfactions for different subscriber types is obvious by the results found by the PRPSO algorithm. Moreover, the characteristics of relationship of operator profit and subscriber satisfaction can be easily observed based on the results. For example, in the *β* = 12 case, the satisfaction is almost the same, even if the profit reaches the maximum value.

### 4.4. Evaluation of Efficiency of Finding Pareto Fronts

Fourth, we evaluate the quality metrics of forming the Praetor front in different number of decision variables. The quality metrics are (1) the average distance of Pareto front and (2) number of points of Pareto front. In general, a smaller average distance indicates that the solutions on the Pareto front are evenly distributed. The average distance is the crowding distance, which is the perimeter of the cuboid formed by using the nearest neighbors as the vertices in the Pareto front; please refer to the paper [5] for more details. The number of points of Pareto front indicates the tractability of the Pareto front for a decision maker. When the number of points or solutions of Pareto front are too large, then the solutions may be intractable for a decision maker.

**Figure 8:**
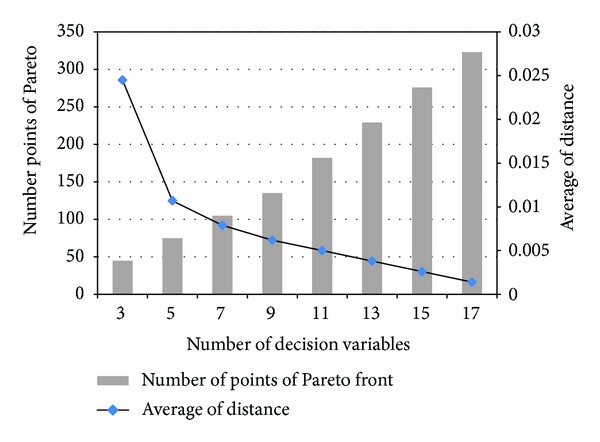
Evaluation of the Pareto front in terms of (1) the number of points of Pareto front and (2) the average of distance, where the right-side *y*-axis is the average distance of the Pareto front, the left-side *y*-axis is the number of points of the Pareto front, and the *x*-axis is the number of decision variables, which is the number of prices (or services).

As shown in Figure 8, the number of points of the Pareto front is increased, but the average distance is decreased, when the number of decision variables is increased. It implies that more points are included in the Pareto front, when the number of decision variables is larger. Selecting a pricing solution from a larger set is more intractable for a decision maker facing higher numbers of price variables. Hence, the decision maker needs to carefully make decisions when they face a higher number of price variables.

## 5. Conclusions

The operators invest huge funds for acquiring the spectrum resources in the LTE network. The operator profit and the subscriber satisfaction are two most important factors. Thus, it is necessary to consider the operator profit factor and subscriber satisfaction factor for pricing resources in the LTE networks. However, most of existing studies only consider the problem about maximizing operator profit. This paper investigates the pricing resources with profit and satisfaction optimization (PRPSO) problem in the LTE network to simultaneously maximize the operator profit and subscriber satisfaction. This paper contributes a theoretical framework to help decision makers in pricing resources, based on the heuristic optimization algorithm—PRPSO algorithm. Compared with the algorithm only solving a single pricing optimization goal, the PRPSO algorithm solves the optimal problem with the consideration of two important goals, which is more helpful for making decisions in pricing.

The PROSO algorithm has been verified and tested by the simulations on the basis of convergence and diversity performance metrics to guarantee the quality of optimal solutions found. The simulation results show that the difference of satisfactions for different subscriber types is obvious. Moreover, the characteristics of relationship of the operator profit and the subscriber satisfaction can also be easily observed based on the results.

## Figures and Tables

**Algorithm 1:**
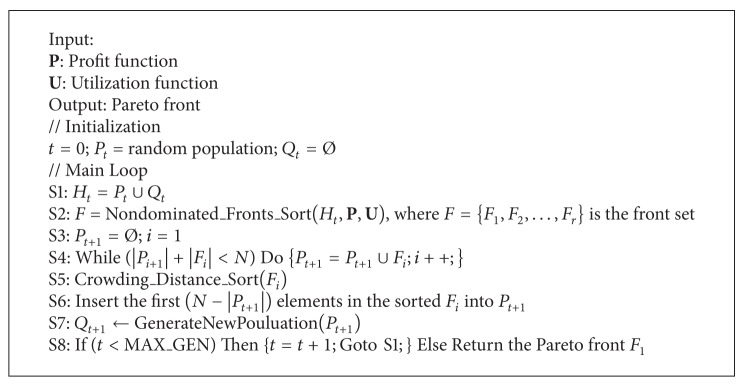
Pricing resources with profit and satisfaction optimization (PRPSO) algorithm.

**Table 1:** Parameter setting.

Parameter	Values
Number of prices (services)	|*X*|, where |*X*| is 3,5, …, 17
Initial population	Uniform random distribution
Population size	15 · |*X*|
Range of price variable (*X*)	(1, 2)
Pareto fraction	0.35
StallGenLimit	100
Toleratethreshold	1 × 10^−6^
MAX_GEN	200 · |*X*|
